# Timing of the Infancy-Childhood Growth Transition in Rural Gambia

**DOI:** 10.3389/fendo.2020.00142

**Published:** 2020-03-24

**Authors:** Robin M. Bernstein, G. Kesler O'Connor, Eric A. Vance, Nabeel Affara, Saikou Drammeh, David B. Dunger, Abdoulie Faal, Ken K. Ong, Fatou Sosseh, Andrew M. Prentice, Sophie E. Moore

**Affiliations:** ^1^Growth and Development Lab, Department of Anthropology, University of Colorado, Boulder, CO, United States; ^2^Institute of Behavioral Science, University of Colorado, Boulder, CO, United States; ^3^Laboratory for Interdisciplinary Statistical Analysis (LISA), Department of Applied Mathematics, University of Colorado, Boulder, CO, United States; ^4^Department of Pathology, University of Cambridge, Cambridge, United Kingdom; ^5^MRC Unit The Gambia, London School of Hygiene and Tropical Medicine, Banjul, Gambia; ^6^Department of Pediatrics, University of Cambridge School of Clinical Medicine, Cambridge, United Kingdom; ^7^MRC Epidemiology Unit, University of Cambridge School of Clinical Medicine, Cambridge, United Kingdom; ^8^Department of Women and Children's Health, King's College London, London, United Kingdom

**Keywords:** infancy, childhood, growth, infancy-childhood transition, hormonal growth regulation

## Abstract

The Karlberg model of human growth describes the infancy, childhood, and puberty (ICP) stages as continuous and overlapping, and defined by transitions driven by sequential additional effects of several endocrine factors that shape the growth trajectory and resultant adult size. Previous research has suggested that a delayed transition from the infancy to the childhood growth stage contributes to sub-optimal growth outcomes. A new method developed to analyze the structure of centile crossing in early life has emerged as a potential tool for identifying the infancy-childhood transition (ICT), through quantifying patterns of adjacent monthly weight-for-age z-score (WAZ) deviation correlations. Using this method, the infancy-childhood transition was identified as taking place at around 12 months of age in two cohorts of UK infants. Here, we apply this method to data collected as part of a longitudinal growth study in rural Gambia [the Hormonal and Epigenetic Regulators of Growth, or HERO-G study, *N* = 212 (F = 99, M = 113)], in order to identify the ICT and assess whether timing of this transition differs across groups based on sex or birth seasonality. We calculated Pearson correlation coefficients for adjacent monthly WAZ score deviations. Based on the patterns of change in the correlation structure over time, our results suggest that the infancy-childhood transition occurs at around 9 months of age in rural Gambian infants. This points to an accelerated ICT compared to UK infants, rather than a delayed ICT. A comparatively later transition, seen in UK infants, allows maximal extension of the high rates of growth during the infancy stage; an earlier transition as seen in Gambian infants cuts short this period of rapid growth, potentially impacting on growth outcomes in childhood while diverting energy into other processes critical to responses to acute infectious challenges. Growth in later developmental stages in this population offers an extended window for catch-up.

## Introduction

The human growth trajectory is complex compared to other large-bodied mammals; passing through infancy, childhood, juvenility, and adolescence before reaching adulthood (1) it is relatively prolonged via the extension of the pre-pubertal period (2), and is best described as sinuous and containing periods of both relatively slow growth and accelerated growth. Earlier perspectives held that certain stages of growth are uniquely derived in humans (e.g., childhood), and the insertion of these stages into an ancestral primate growth trajectory are responsible for the prolongation of growth overall in humans—itself hypothesized to permit a longer period of brain development (3), to build metabolic and cognitive capital to use in later life (4), or to increase chances of success in relationship to extrinsic mortality risk both in adulthood and during immaturity (5, 6). More recent analyses have suggested that while the human growth trajectory is indeed extended relative to that of our closest living relatives, those stages previously considered novel are present in other non-human primate species, and relatively accelerated, and/or compressed in their time course of expression (7–12). While differences in the length of each stage and the nature of their transitions can be influenced by evolutionary relationships, how quickly or slowly individuals pass through growth stages can also be attributed to a number of (not mutually exclusive) factors, including nutritional status, morbidity burden, and aspects of the social and physical environment such as dominance rank, mortality risk, and seasonality (13–15). In humans, there is significant inter- and intra-population variation in the timing of maturational events, and rates of growth (16, 17). From an applied standpoint, growth in early life has been linked to developmental and health outcomes across the life course such as childhood obesity (18); childhood adiposity; and age at menarche (19).

The model that has been most frequently used to identify the infancy-childhood growth transition (ICT) is the infancy-childhood-puberty (ICP) growth model of (20), which centered the identification of human growth transitions within the context of the hormonal regulation of growth. The three identified phases of growth are described as overlapping and continuous, in that the physiological regulation of each stage “layers” with time and summarily contribute to the growth patterns seen during each stage as well as the ultimate outcome (adult stature). In this model, the infant stage of growth is described as a decelerating continuation of the fetal growth trajectory, largely under the control of insulin and the insulin-like growth factors as mediators of nutritional status. Karlberg proposed that the childhood transition, where the rapidly decelerating growth of infancy switches over to the steadier state and growth rate plateau of childhood, is initiated by the endogenous regulation of growth hormone (GH), starting toward the end of the first year of life, and signaled by the so-called infancy-childhood spurt (ICS). During the puberty phase of Karlberg's model, the hypothalamic-pituitary-gonadal axis further modifies the pulsatile release of GH, and the pubertal growth spurt brings an individual to their final adult stature.

From a clinical perspective, this model is important because it emphasizes the continuity of the growth process and the multiple physiological inputs in shaping growth patterns over time (although these are necessarily still simplified). Understanding variation in the timing of transitions, and underlying causes, could be of great import if it could be demonstrated that this variation is related to factors that might be modified to positively affect growth, particularly before an individual has found the growth trajectory that they will track, or their growth “canal” (21). In addition, the relationship of the timing of the ICT to the presence or absence of catch-up growth in infancy and childhood could be important for understanding not only growth outcomes but also associated health-related outcomes (18). It has been proposed that individuals who experience adverse conditions in early life have insufficient energetic reserves necessary for the ICS, and so the transition to childhood is delayed [DICT, (22)]. Alternatively, in the context of infancy as a phase of rapid growth where growth rate is dependent on nutritional status, it is possible that a truncated period of infancy and an earlier onset of childhood could represent an adaptive developmental response in the case of nutritional insufficiency, or of frequent morbidities directing the investment of energetic resources away from growth and toward immune function and repair. Although body growth rates are lower during childhood than infancy, metabolic costs associated with brain maturation reach a peak in middle childhood (23); by implementing a low-cost somatic growth strategy during childhood, and/or by cutting short infancy and shifting into childhood earlier in development, an individual may free up more capital to support the expensive development of the brain. In the short-term, this could result in outcomes such as stunting; however, later phases of rapid development (i.e., adolescence) may offer opportunities for catching up before the attainment of final adult height.

The ICP model has been implemented in the majority of ICT studies to date, but this method is not without its drawbacks. Perhaps most importantly, although growth data are modeled using the ICP, the ICT itself is identified through visual inspection of plotted growth curves. While previous studies have reported a high degree of interobserver agreement using this method (24), it remains a subjective approach. Importantly, a short-term acceleration in growth velocity just prior to the onset of childhood is the basis of both the (visual) identification of the transition itself, and the framework for understanding the effects of a DICT. The ICT/ICP method has been used in clinical contexts to understand the relationship of a delay in the transition to growth outcomes. For example, in one study of children with idiopathic short stature (ISS), a DICT of around 4 months corresponded to a significantly reduced growth rate during the first 2 years of life (25). The aim of this particular study was to determine the optimal timing of GH therapy in order to maximize outcomes; however, it should be emphasized that the growth patterns of these children, although not necessarily coupled with hormone dysfunction, cannot be taken as representative of broader growth patterns within the larger population from which they were drawn.

This quasi-pathological perspective is found much of the literature on ICT/DICT, yet it is offered as a metric that might be applied to help understand “…the main mechanism resulting in short stature in children living in poor areas of developing countries (p. 6, 21).” This proposes ICT as plastic and significantly influenced by environment; but, other studies suggest a relatively low contribution of the external environment to the timing of the transition [i.e., 28% total variance explained, (26)], and the importance of genetic factors [i.e., mid-parental height, (24)]. Some of these inconsistencies may result from the conflation of the ICT with the ICS, and the associated attempts to disentangle genetic vs. environmental effects on a developmental event (ICS) vs. a transition phase (ICT). One longitudinal study of the ICS in Dutch infants confirmed a transient growth acceleration at around 9 months of age, although the authors caution that this does not justify the construction or use of the ICP as a reference (27). Additionally, the frequency of anthropometric measurements used in ICT studies to date ranges between eight times within the first year of life (24) to nine times within the first 3 years of life (25); this raises questions regarding the minimum number of measurements needed to confidently identify transient growth accelerations in early life.

In 2016, Cole and colleagues published a study that tracked weight centile crossing in UK infants, using two large longitudinal cohorts [Widdowson and Cambridge Infant Growth Study (CIGS), (28)]. The aim of this study was to better understand how previous weight centile crossing predicts future weight gain, and as part of this aim the investigators characterized the correlation structure of monthly weight for age z-score (WAZ) deviation (i.e., centile crossing) by assessing whether the direction of month-to-month change in WAZ was influenced by the direction of WAZ change in a prior time interval. Through application of this method, they identified two main and sequential patterns of growth feedback in infants in both of the UK cohorts: positive feedback (represented by positive correlations between successive deviations, indicating similar direction of centile crossing in each pairs of months analyzed) during the first few months, followed by negative feedback (indicated by negatively correlated successive deviations, and illustrative of growth moving in different directions across the pairs of months analyzed) in the last half of the first year of life. These complementary modes of feedback (28), propose, work together to canalize growth within a particular range of centiles, and awareness of this underlying structure is important for clinicians who are trying to predict and evaluate growth. Following the period of negative feedback in the latter half of the first year of life, the authors further propose that an additional shift in the correlation structure—when the correlations between adjacent monthly WAZ deviations break free of the negative correlation/negative feedback loop and approaches zero—could represent a shift from the infancy stage of growth to the childhood stage of growth (28). In both UK infant cohorts, correlation coefficients between adjacent monthly WAZ deviations were most strongly positive between 3 and 4 months of age (*R* = 0.3), and then decreased reaching a nadir at around 10–11 months (*R* = −0.3); at around 12 months, correlations were close or projected to be close to zero, and this was suggested by the authors as possibly being indicative of the ICT. Using this method, the ICT is identified as taking place later than previously identified in other cohorts from similar populations, using length and the ICP model (~12 vs. ~9 months, reviewed above). The authors note that their findings and implications may not apply in populations with higher frequencies of growth disruption due to chronic infection or malnutrition.

Here, we use the approach described by Cole et al. (28), and summarized above, to identify the ICT in a cohort of Gambian infants based on detailed longitudinal weight growth data. Our aims are to both build context for understanding variation in this transition and to specifically investigate the timing of the transition in relationship to infant sex and birth seasonality. The infants in this study live in the West Kiang region of The Gambia, a rural subsistence farming community of savanna and farmland. The annual wet season, from July to October, is characterized by a decline in food stores, an increase in physical labor associated with farming, and a rise in morbidities such as malaria, bacterial infections, and environmental enteropathy especially affecting children under 3 years of age. The dry season, from November to June, brings an increased food supply, less physical labor, and lower rates of morbidity. These factors contribute to variation in growth patterns and health outcomes in individuals living in this region (29–31). Based on prior ICT studies, we hypothesize that Gambian infants should show a delayed ICT compared to UK infants; because our analysis is limited to the first year of life, we specifically hypothesize that we should not be able to identify an ICT in our sample if it is delayed relative to UK infants (where the ICT was identified at ~12 months). We further hypothesize a seasonal effect on the timing of the ICT.

## Materials and Methods

### The HERO-G Study

This study uses observational data collected as part of a longitudinal cohort study looking at the Hormonal and Epigenetic Regulators of Growth (HERO-G). The primary focus of HERO-G is infant growth from birth to 2 years of age; data were recorded every other day for the first 12 months and additional measurements were recorded at 18 and 24 months. The full HERO-G protocol is described elsewhere (32). The data included in this analysis are limited to the first year of life because it is focused on monthly deviations. To include the 18 and 24-months data would require interpolation to derive monthly values across the second year of life, and this is not warranted or appropriate for this particular analytical approach. Ethical approval for the study was given by the joint Gambia Government/Medical Research Council (MRC) Unit The Gambia Ethics Committee (SCC 1313v3), with additional approval from the University of Colorado Institutional Research Board (protocol number 13-0441). Prior to the start of the study, community approval was obtained from each participating village, and written, informed consent was obtained from each participating family.

### Infant Anthropometry

Following their naming ceremony at 1 week of age, infants were seen every other day in their home village for anthropometric measurements until they reached 1 year of age. At each home visit, field workers measured infant weight according to standard protocols. Infants were undressed and weighed using a Seca 336 digital weighing scale. Weights were recorded to the nearest 10 g. Scales were calibrated each day prior to measurement, and weights were recorded in triplicate.

### Data Treatment and Analysis

Our analysis follows that of Cole et al. (28), wherein we examine patterns of monthly WAZ deviation correlations in order to identify shifts in patterns of correlation (i.e., negative and positive feedback) as well as determine at what point these feedback patterns indicate a shift to a new phase of growth (ICT) as correlation coefficients approach zero following the lowest negative correlation value (nadir). We used the average of our triplicate weight measurements collected every other day, after removing within-day and between day outliers. We define within-day outliers as any measurement which is farther than 0.15 kg away from the other two measurements. If no two measurements are within 0.15 kg of each other, we do not use data from that day. We define between day outliers as any day where the average weight is >1 kg different from the average of the weight on the neighboring days. Weights were converted to age- and sex-adjusted z-scores using WHO growth standards and references (33, 34).

After removing outliers, we had data coverage sufficient for this analysis from 212 infants (99 female, 113 male). We utilized the increased measurement frequency of our data by using averages of 7-days windows. Specifically, we filtered the data down to 12 adjacent monthly 7-days windows. The windows were centered at the following ages (in days), 12 + i(30), i = 0,…,11. We then computed the average WAZ (z_i_), which were calculated as the average of the z scores in the ith 7-days window for each individual. Next, we computed the deviations (d_i_), which are the difference in adjacent z_i_ and are defined by,

$$\begin{array}{l} d_{i}=z_{i}-z_{i-1}\;i\in \left(1,2,\ldots ,12\right) \end{array}$$

Finally, to test the significance of the correlation between adjacent d_i_ we used the R function cor.test (35). The cor.test function computes the Pearson correlation coefficient using all pairwise complete pairs of the data and then performs a one sample *t*-test to determine whether or not the correlation is significantly different than 0. The Pearson correlation (r_i,i−1_) coefficient between adjacent deviations d_i_ and d_i−1_ is defined by,

$$\begin{array}{l} r_{i,i-1}=\frac{\sum_{k=1}^{n}\left(d_{i,k}-\;\bar{d_{i}}\right)\left(d_{i-1,k}-\;\bar{d_{i-1}}\right)}{\sqrt{\sum_{k=1}^{n}{\left(d_{i,k}-\;\bar{d_{i}}\right)}^{2}}\sqrt{\sum_{k=1}^{n}{\left(d_{i-1,k}-\;\bar{d_{i-1}}\right)}^{2}}} \end{array}$$

The Pearson correlation coefficients r_i,i−1_ and associated *p*-values reported in this paper were all computed using the cor.test function. In all figures the correlations are plotted at the midage of the two periods over which the value is computed. We calculated correlations for (1) all individuals, (2) female and male infants separately, and (3) wet (June through October) and dry (November through May) season births separately.

## Results

### Summary Statistics

**Figure 1:**
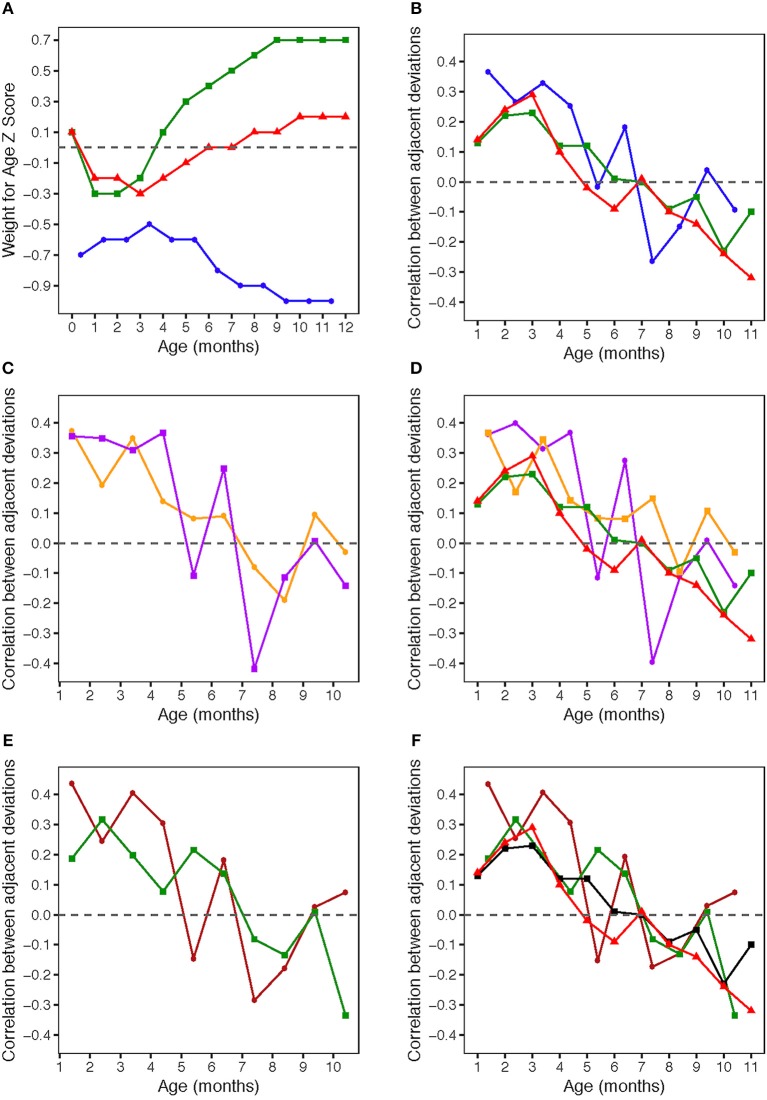
**(A)** Weight for age z-scores (WAZ) by age. WAZ means and standard deviations given in Table 1. Blue circles: HERO-G infants; Green squares: Widdowson cohort; Red triangles: CIGS cohort. Widdowson and CIGS data from Cole et al. (28). **(B)** Correlations by age between adjacent pairs of WAZ deviations. Correlations were computed using data from every available subject. The sample sizes for each correlation are given in Table 2. Blue circles: HERO-G infants; Green squares: Widdowson cohort; Red triangles: CIGS cohort. Widdowson and CIGS data from Cole et al. (28). **(C)** Correlations by age between adjacent pairs of WAZ deviations. The sample sizes for each correlation are given in Table 2. Orange circles: female HERO-G infants; Purple squares: male HERO-G infants. **(D)** Correlations by age between adjacent pairs of WAZ deviations. Orange circles: female HERO-G infants; Purple squares: male HERO-G infants; Green squares: Widdowson cohort; Red triangles: CIGS cohort. Widdowson and CIGS data from Cole et al. (28). **(E)** Correlations by age between adjacent pairs of WAZ deviations. The sample sizes for each correlation are given in Table 2. Brown circles: HERO-G infants born in the dry season; Green circles: HERO-G infants born in the wet season. **(F)** Correlations by age between adjacent pairs of WAZ deviations. Brown circles: HERO-G infants born in the dry season; Green circles: HERO-G infants born in the wet season; Green squares: Widdowson cohort; Red triangles: CIGS cohort. Widdowson and CIGS data from Cole et al. (28).

Summary statistics for Gambian monthly WAZ and monthly changes in WAZ are shown in Table 1, together with the same data from the UK cohorts (from 26), and patterns of the three groups are illustrated in Figure 1A. In Gambian infants, the mean WAZ increases during the first few months of life (albeit a slight increase on a negative WAZ value), at the same time that UK infant WAZ decreases. Similarly, WAZ decreases following ~4 months of age in Gambian infants while it increases at the same time period in UK infants. When all Gambian subjects are combined across categories of sex and season of birth, the mean WAZ increases from −0.7 in month 1 to −0.6 in months 2 and 3, and −0.5 in month 4. It stays close to this value in months 5 and 6 (−0.6), and then declines starting at 7 months of age, down to −1.0 at 10–12 months of age. The mean change in WAZ was positive for the first month but zero for months 2–4, negative in months 5–10, and back to zero at month 11. The SD for monthly changes in WAZ maintained at 0.3 for most months (0.4 in month 1). In Gambian infants, we see consistent moderate variation across the first year of life, whereas there was more variation in 0–6 months compared to 6–12 months in UK infants. Compared to the Widdowson and CIGS results, the HERO-G WAZ scores are lower, and the WAZ deviations and WAZ deviation SDs are smaller.

**Table 1:** Average weight z score (WAZ) and average WAZ deviations by months, comparison between HERO-G and Widdowson and CIGS cohort data, reproduced from ^a^Cole et al. (28).

**HERO-G data**					**Widdowson data**^****a****^			**CIGS data**^****a****^	
**Age months**	***n***	**Weight *z* score**	**Weight *z* score deviation**	***n***	**Weight *z* score**	**Weight *z* score deviation**	***n***	**Weight *z* score**	**Weight *z* score deviation**
1	194	−0.7 ± 0.9	0.1 ± 0.4	1,040	0.1 ± 1.1	−0.4 ± 0.6	253	0.1 ± 1.0	−0.3 ± 0.6
2	198	−0.6 ± 0.9	0.0 ± 0.3	1,080	−0.3 ± 0.9	0.0 ± 0.4	254	−0.2 ± 0.9	−0.1 ± 0.4
3	201	−0.6 ± 1.0	0.0 ± 0.3	1,079	−0.3 ± 1.0	0.2 ± 0.4	255	−0.2 ± 0.9	0.0 ± 0.3
4	192	−0.5 ± 1.0	0.0 ± 0.3	1,072	−0.2 ± 0.9	0.2 ± 0.3	253	−0.3 ± 0.9	0.1 ± 0.3
5	193	−0.6 ± 1.0	−0.1 ± 0.3	1,071	0.1 ± 0.9	0.2 ± 0.3	251	−0.2 ± 0.9	0.1 ± 0.3
6	197	−0.6 ± 1.0	−0.1 ± 0.3	1,065	0.3 ± 0.9	0.1 ± 0.2	251	−0.1 ± 0.9	0.1 ± 0.2
7	194	−0.8 ± 1.0	−0.1 ± 0.3	1,054	0.4 ± 0.9	0.1 ± 0.2	247	0.0 ± 0.9	0.0 ± 0.2
8	192	−0.9 ± 1.0	−0.1 ± 0.3	1,043	0.5 ± 0.9	0.1 ± 0.2	251	0.0 ± 0.9	0.1 ± 0.2
9	186	−0.9 ± 1.0	−0.1 ± 0.3	1,009	0.6 ± 0.9	0.1 ± 0.2	250	0.1 ± 0.9	0.0 ± 0.2
10	182	−1.0 ± 1.0	−0.1 ± 0.3	969	0.7 ± 0.9	0.0 ± 0.2	247	0.1 ± 0.9	0.1 ± 0.2
11	187	−1.0 ± 0.9	0.0 ± 0.3	913	0.7 ± 0.9	0.0 ± 0.2	245	0.2 ± 0.9	0.0 ± 0.2
12	187	−1.0 ± 0.9	NA	852	0.7 ± 0.9	0.0 ± 0.2	247	0.2 ± 0.9	0.0 ± 0.2

### Correlation Patterns—Entire Dataset

Figure 1B shows the correlations between pairs of monthly WAZ deviations during the first year in all HERO-G infants together. The correlations are plotted (for all figures) at the middle of the two adjacent periods, meaning the correlation between deviations d_i_ = z_i+1_ − z_i_ and d_i+1_ = z_i+2_ − z_i+1_ is plotted at the center of the window over which z_i_ was calculated. The correlations for adjacent monthly deviations start at 0.37 at 1 month, decrease to 0.27 at 2 months, increase slightly to 0.33 at 3 months, then decrease back to 0.25 at 4 months (Table 2). These correlations are all significant (*P* < 0.001). At 5 months, the correlations decrease sharply but then rebound at 6 months to 0.18 (*P* < 0.05). Following this the correlations plummet to the nadir of −0.27 at 7 months (*P* < 0.001). After the nadir, correlations increase to −0.2 at 8 months (*P* < 0.05), and rise to just above zero (0.04, ns) at 9 months, after which they decrease again to −0.09 (ns). The HERO-G deviation correlation pattern differs from that seen in the Widdowson and CIGS cohorts in a few ways. First, the peak correlation coefficient is at the earliest time point (1 month) in Gambian infants, vs. 3 months in UK infants. In relationship to this, there is a clear and consistent pattern of increasing correlations across the first 3 months in UK infants, whereas correlations decrease then increase in Gambian infants across this same time period. Second, while there are clear periods of ‘catch-up' (positive feedback—birth to 3 months) and ‘catch-down' (negative feedback −4-11 or 12 months) in the UK infants, as defined by Cole et al. (28), there are no such clear corresponding phases in the Gambian infant pattern. Instead, there are several shorter-term shifts from positive to negative feedback, although the overall direction of the correlations (moving from positive to negative values over time), and the range of correlation coefficients themselves is similar. Third, the correlation coefficient approaches zero at different ages; based on the full Gambian dataset, this takes place around 9 months of age, and in the Widdowson cohort this takes place closer to 11 months. This shift is not yet seen in infants in the CIGS cohort although (28) estimate that this would occur around 12 months of age.

**Table 2:** Correlations by age between adjacent pairs of weight z score deviations measured over 30 days and associated *p*-values for the full HERO-G dataset analyzed and for each subset of the data considered.

	**All (*****n*** **=** **212)**		**Female (*****n*** **=** **99)**		**Male (*****n*** **=** **113)**		**Born in dry season (*****n*** **=** **138)**		**Born in wet season (*****n*** **=** **74)**	
**Age (months)**	**Corr**	***p*-value**	**Corr**	***p*-value**	**Corr**	***p*-value**	**Corr**	***p*-value**	**Corr**	***p*-value**
1	0.37	0.0000	0.37	0.000	0.36	0.000	0.44	0.00000	0.19	0.133
2	0.27	0.0002	0.19	0.067	0.35	0.000	0.24	0.00599	0.32	0.009
3	0.33	0.0000	0.35	0.001	0.31	0.001	0.41	0.00000	0.20	0.118
4	0.25	0.0004	0.14	0.205	0.37	0.000	0.30	0.00055	0.08	0.543
5	−0.02	0.8132	0.08	0.460	−0.11	0.276	−0.15	0.10052	0.22	0.092
6	0.18	0.0119	0.09	0.399	0.25	0.012	0.18	0.03886	0.14	0.290
7	−0.27	0.0002	−0.08	0.446	−0.42	0.000	−0.29	0.00109	−0.08	0.532
8	−0.15	0.0472	−0.19	0.084	−0.11	0.269	−0.18	0.04822	−0.13	0.324
9	0.04	0.5981	0.09	0.394	0.01	0.949	0.03	0.78016	0.01	0.949
10	−0.09	0.2212	−0.03	0.791	−0.14	0.174	0.07	0.42418	−0.34	0.010

### Correlation Patterns—Infant Sex

When separated by infant sex (female *n* = 99, male *n* = 113), different patterns of WAZ deviation correlations in female and male infants become apparent (Figure 1C). Female infant WAZ deviation correlations shift during the first few months of life, from a peak zenith of 0.37 at 1 month, to 0.2 at 2 months, to 0.35 at 3 months. At 4 months, the deviation correlation drops to 0.15, and stays around 0.1 through months 5 and 6 before dropping below zero at 7 and 8 months. The nadir for female correlations is −0.2 at 8 months, and increases to 0.1 at 9 months, finally dropping down again to just below zero at 10 months. Only months 1 and 3 of the female deviation correlations are significant (*P* ≤ 0.001). The male pattern differs from the female pattern in a few respects. Deviation correlations remain near constant during the first 4 months (0.36, 0.35, 031, 0.37, all significant at *P* ≤ 0.001), reaching a zenith at 4 months. Across this same time period, female deviation correlations shift several times (Figure 1C). Male deviation correlations drop precipitously at 5 months to −0.11, and rebound to 0.25 at 6 months (*P* < 0.05). Female values remain fairly constant during the same time period. The male nadir (−0.42) is reached at 7 months (*P* < 0.001), and correlations move in a positive direction in months 8 (−0.11) and 9 (0.01), where it approaches zero (0.01) before dropping down to −0.14 at 10 months. The female nadir (−0.19) is reached shortly after 8 months, and then the correlation pattern tracks that of males in months 9–10. Overlaying male and female Gambian infant patterns on those of UK infants (28) illustrates that while the overall direction of deviation correlations over time is similar across all groups, the female HERO-G infant pattern and range of correlation coefficients is more similar to those of UK infants than the male HERO-G infant pattern (Figure 1D), especially between months 4–7.

### Correlation Patterns—Infant Season of Birth

Deviation correlations computed separately for infants born during the dry season (*n* = 138) and the wet season (*n* = 74) are plotted in Figure 1D. Several differences are apparent in the pattern of correlations between birth seasons. From 1 to 2 months, infants born in the wet season initially catch up (0.19 to 0.32), while infants born in the dry season initially catch down (0.44 to 0.24), then both shift in opposite directions from 2 to 3 months, with infants born in the dry season catching up (0.24 to 0.41), and those born in the wet season catching down (0.32 to 0.20). All of these correlations between adjacent deviations are significant for infants born in the dry season (*P* < 0.01), while only the correlation illustrating catch-up between months 1 and 2 for infants born in the wet season is significant (*P* < 0.01). Deviation correlations decrease from months 3 to 4 in infants born in both seasons (dry season: 0.41 to 0.30; wet season: 0.2 to 0.08). At 5 months patterns diverge again, with a positive shift for infants born in the wet season (0.22) and a steep drop in infants born in the dry season (−0.15). At 6 months, deviation correlations are very similar for both groups (dry season: 0.18; wet season: 0.14), and both drop below zero at 7 months (dry season nadir: −0.29; wet season: −0.08). Between 8 and 9 months, infants born in both seasons move in a positive direction again (dry season: −0.18 to 0.03; wet season −0.13 to 0.01); at 9 months the deviation correlation for both groups is close to zero. At 10 months, the patterns diverge once again, as deviation correlations for infants born in the dry season increases slightly (0.07), and that for infants born in the wet season drops and reaches its minimum (−0.34). The deviation correlation pattern of HERO-G infants born in the wet season is more similar to that of the Widdowson and CIGS cohorts than HERO-G infants born in the dry season (Figure 1E); patterns of the three groups are almost identical across the first 4 months. The pattern of HERO-G infants born in the wet season diverges between 4 and 6 months, suggestive of positive feedback and catch-up growth, while the CIGS and Widdowson infants continue to catch down during this time. The patterns of all four groups are most similar between 7 and 9 months (Figure 1F). HERO-G infants born during the dry season show a pattern of increasing deviation correlations toward the end of the first year, similar to what is seen in the Widdowson cohort; the deviation correlations in HERO-G infants born in the wet season, like infants in the CIGS cohort, do not increase past the nadir that falls on the last time point.

## Discussion

We analyzed monthly WAZ deviation correlations across the first year of life, applying the method of Cole et al. (28), in order to identify the infancy-childhood transition in rural Gambian infants. Cole et al. (28) identified two ‘phases' of deviation correlations within the first year of infant life: (1) positive feedback during the first few months, and (2) negative feedback from 6 to 10/11 months. These feedback phases were proposed as the mechanism by which an individual finds and tracks their growth canal. Further, Cole et al. (28) proposed that the point after the nadir of the negative feedback phase at which the correlations approach zero could represent a ‘release' from the negative feedback prior to that point, and a shift into a new phase of growth (i.e., childhood via the ICT).

We hypothesized, based on prior work on the ICT using the ICP model, that (1) the ICT in Gambian infants as a group would be delayed compared to the two UK infant cohorts analyzed by Cole et al. (28), and (2) the ICT in Gambian infants born in the wet season would be delayed (DICT) relative to the ICT in infants born in the dry season. The results of our analysis did not support the first hypothesis; based on the ages at which adjacent monthly deviation correlations approached zero following a nadir, the HERO-G infants go through an ICT earlier than either the Widdowson or CIGS cohorts (~9 months of infant age compared to ~12 months).

The pattern of deviation correlations in infants born in the wet season was such that the nadir of the correlations fell on our last calculable correlation (10 months of infant age), so we were unable to determine whether and when after that point deviation correlations might have approached zero.

However, the deviation correlations were close to zero at 9 months of age, the time point prior to the wet season birth nadir, at the same time as they were close to zero in infants born in the dry season. Our next measurement following 12 months of age in the HERO-G infants was 18 months, and interpolation would be required to derive monthly WAZ scores, and calculate deviation correlations; interpolation of monthly values from biannual known data points is not appropriate in this case, and therefore we are unable to further test this hypothesis with data from this cohort. Like infants born in the wet season, the CIGS cohort infants (28) did not show an increase in WAZ deviation correlations following the nadir, although based on later measurements it was concluded that the transition would have likely followed the nadir at around 12 months. It may also be the case that multiple rounds of growth faltering and catch-up in infants born in the wet season would complicate or obscure patterns of deviation correlations calculated with this method. We also did not find any effect of sex on the timing of the ICT, although our patterns of deviation correlations suggest that males have an earlier correlation nadir than females, and take 2 months following the nadir to approach a zero correlation (females take one). The overall pattern of deviation correlations in HERO-G females was more similar to that of UK infants compared to HERO-G males. Only two of the ten female correlations were significant, while six of ten were significant for males; in general, correlations were also higher in males compared to females.

That Gambian infants transition to childhood earlier than UK infants, rather than being delayed, runs counter to previous ICT analyses that use the ICP model to identify the ICT. There are a couple of potential methodological reasons why this might be so. First, we used weight and adjacent monthly deviation correlations of WAZ to identify the ICT, instead of length measurements as used by the ICP model. It is probable that shifts in weight precede shifts in length and as such appear at earlier chronological ages. Cameron (36) notes that weight is a more “eco-sensitive” characteristic than length, since it can change rapidly over short periods of time based on various acute stressors. The linkages between short-term episodes of wasting, and longer-term stunting outcomes were examined in a large (*n* > 5,000 individuals) data set derived from regular growth monitoring of Gambian children (37). Results demonstrated that episodes of wasting were predictive of stunting, and suggest that the outcome of stunting is an adaptive phenotype in the context of episodes of wasting in the first year of life. However, even if a weight-based ICT can be conceived to causally precede a length-based ICT, this would still imply that a length-based ICT in Gambian infants would be proportionally advanced relative to a length-based ICT in UK infants. Second, the diagnostic indicator of an ICT using the ICP model is a transient acceleration in length velocity, identified through visual inspection of individual growth curves. The basis for using the ICS as a marker for the ICT is the proposal that an infant must have a certain amount of energetic reserve to transition into childhood from infancy, and without it the infancy-childhood spurt is not possible, causing the transition to be delayed. Using this method, studies have identified an ICS and the ICT in infants who have had as few as four measurements taken during the first year of life; this raises questions about whether the ICS is a real biological phenomenon or an artifact of the ICP model parameters. This possibility notwithstanding, it is interesting to note that in the full HERO-G dataset, as well as that calculated separately for males and infants born in the dry season, WAZ deviation correlations show a brief rebound pulse at 6 months of age, crossing from a negative to positive correlation before shifting negatively again. This might represent a transient acceleration in weight, potentially preceding a transient acceleration in length.

More generally, acceleration in developmental timing is just as plausible a response to adverse environmental conditions as a delay; developmental rate and mortality risk interact and form the basis for individual trade-offs between survival, immune function, physiological efficiency, and so on, as individuals navigate their course to maturity (38). Life history theory enumerates the many potential contributors to shaping an individual's life course, including the rate of development and timing of developmental events. Growth rate and growth duration are linked to age at first reproduction. Growing for a longer period of time and ending up as a larger-bodied adult with a later age at reproductive maturation, is one way to achieve higher fertility and decreased mortality of offspring for populations with low sources of extrinsic mortality. Ceasing growth early, and initiating reproduction at a smaller adult size, offers an advantage for high-mortality environments in that it provides a better chance at reproducing prior to death. Therefore, population- and species-level differences in growth patterns and resulting terminal size are shaped by past and current environments, with both earlier- and later-maturing pathways having their respective costs and benefits. A large body of literature discusses accelerated development as an adaptive response to indicators of a high-mortality risk environment. This has been shown in animals ranging from insects (39) to primates (40). In humans, a short stature phenotype seen in several populations globally (e.g., the Aeta, Agta, Batek, Biata) has been proposed to result from a shortened growth period of slower growth, facilitating earlier reproductive maturation in high mortality-risk environments (41). Conversely, previous research in The Gambia has demonstrated that taller mothers—indicative of a longer period of growth—have lower offspring mortality (42). Other authors have suggested that the tempo of maturation is shaped by two key transitions: the ICT, which sets the pace for height, and the childhood-juvenility transition (the ICP model subsumes juvenility within childhood), which offers another opportunity for individuals to recalibrate their tempo based on environmental influences during that time (43). Taken together, this body of work suggests that it is possible that in populations that have experienced challenging environments/stressors in early life for generations, accelerating the age at onset of childhood is an adaptive tradeoff that effectively allows an individual to conserve resources to be channeled to brain development during this critical phase of development, while somatic growth deficits can be caught up later in growth. Recent analysis of the full course of development in The Gambia has demonstrated catch-up growth during an extended adolescence can ameliorate much of the deficit incurred via growth stunting by 2 years of age, especially in girls (44).

In summary, using a method to identify the ICT based on WAZ deviation correlations within the first year of life, we have identified this transition to take place at around 9 months of age in the HERO-G cohort of rural Gambian infants. This is approximately 3 months earlier than the ICT was suggested to take place in two cohorts of UK infants, based on the same method. This suggests that the ICT in Gambian infants is accelerated, instead of delayed as would be predicted based on previous analysis of the ICT using the ICP model (20). Further, we found different patterns of age-related correlations across groups split by sex and season of birth, suggesting that this approach might be used in other populations to better understand how different intrinsic and extrinsic factors shape the pattern of correlations across the first year of life. Ultimately, a truncated infancy growth period and an associated accelerated timing of the ICT may be linked to a trade-off for Gambian infants, whereby cutting short the high growth-rate, high-cost infancy stage, and transitioning to childhood earlier permits allocation of resources to brain development without simultaneous drain of these resources by rapid body growth.

## Data Availability Statement

The raw data supporting the conclusions of this article are stored on the Open Science Framework (OSF), doi: 10.17605/OSF.IO/5ND3Y, and at the time of article submission are available on request and subject to review. These data will be made publicly available no later than July 1, 2021. Requests to access the datasets should be directed to the corresponding author.

## Ethics Statement

The studies involving human participants were reviewed and approved by the joint Gambia Government/MRC Unit The Gambia Ethics Committee (Project number SCC1313v3) University of Colorado Boulder Institutional Review Board (protocol number 13-0441). Written informed consent to participate in this study was provided by the participants' legal guardian/next of kin.

## Author Contributions

RB, NA, DD, KO, AP, and SM conceived of and designed the HERO-G study. SD supervised and coordinated field staff. AF organized and maintained the study database. FS supervised ultrasonography and midwifery. GO and EV performed the statistical analysis. RB wrote the first draft of the manuscript. All authors contributed to manuscript revision, read, and approved the submitted version.

### Conflict of Interest

The authors declare that the research was conducted in the absence of any commercial or financial relationships that could be construed as a potential conflict of interest.
